# Supplementary material for: Substantial underestimation of SARS-CoV-2 infection in the United States
Source: Nat Commun. 2020 Sep 9;11:4507. doi: 10.1038/s41467-020-18272-4 (PMC7481226; doi:10.1038/s41467-020-18272-4)
Supplement: Supplementary file 1 — Supplementary Information [file 41467_2020_18272_MOESM1_ESM.pdf]

Supplementary Information

Substantial underestimation of SARS-CoV-2 infection in the United States

Sean L. Wu, Andrew Mertens, Yoshika S. Crider, Anna Nguyen, Nolan N. Pokpongkiat, Stephanie Djajadi, Anmol Seth, Michelle S. Hsiang, John M. Colford Jr., Art Reingold, Benjamin F. Arnold, Alan Hubbard, Jade Benjamin-Chung

Supplementary Figure 1. Distribution of expected SARS-CoV-2 infections by state correcting for bias due to incomplete testing and imperfect test accuracy

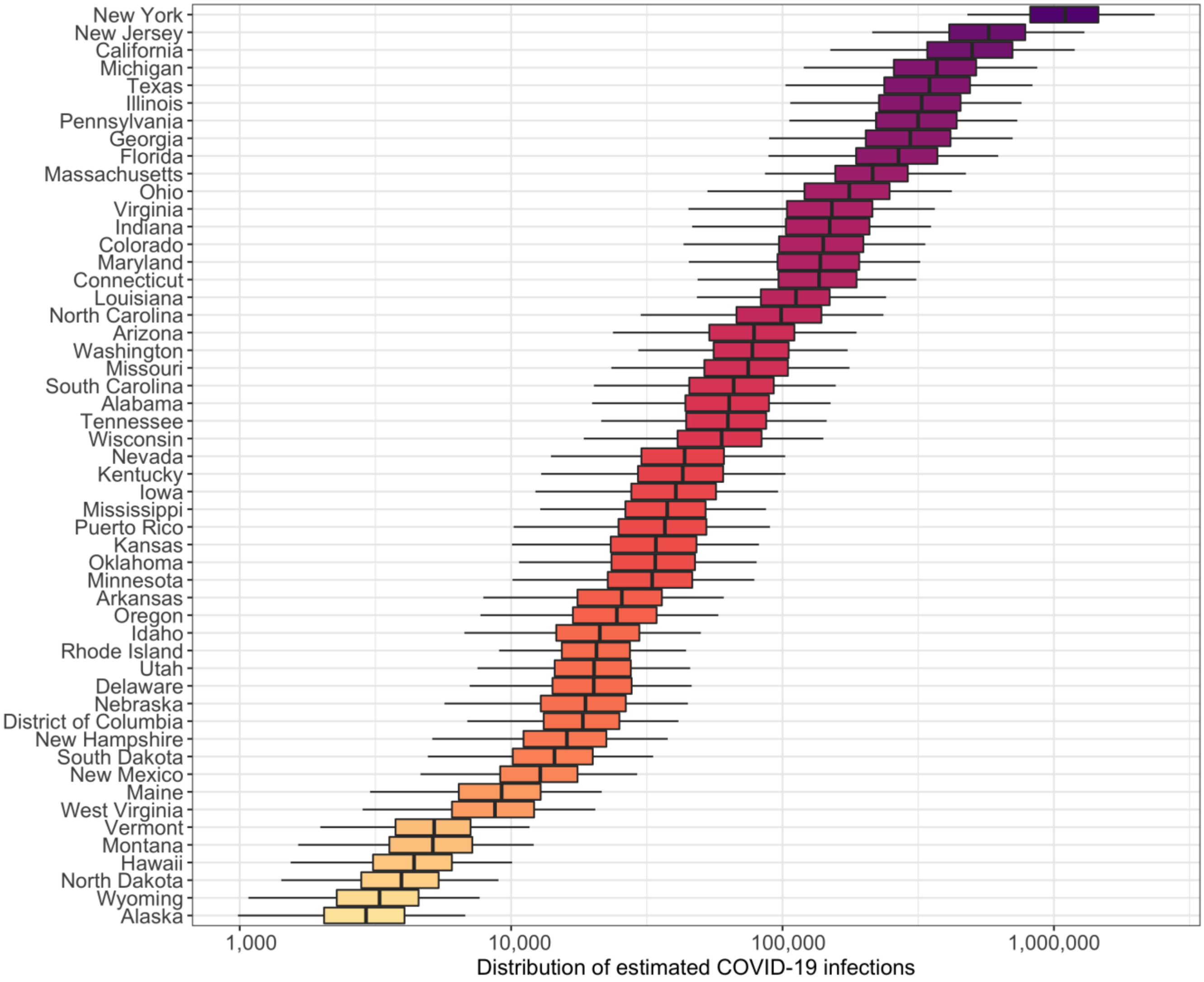

Analyses include cumulative confirmed COVID-19 case counts up to April 18, 2020. Estimated infections were from a Bayesian probabilistic bias analysis to correct for incomplete testing and imperfect test accuracy; for each state we drew 10^4 Monte Carlo samples from the distribution of estimated SARS-CoV-2 infections. Each box plot is colored by $\log_{10}$(median of sampled infections for each state) and summarizes the simulated bias-corrected cumulative infections for each state, such that warmer colors correspond to lower values and cooler colors to higher values. The lower and upper whiskers of the boxplots correspond to the 0.025 and 0.975 quantiles, respectively, and the lower and upper hinge to the 0.25 and 0.75 quantiles. The heavy vertical line (centre) is the median of sampled values.

Supplementary Figure 2. Results of Sensitivity Analysis

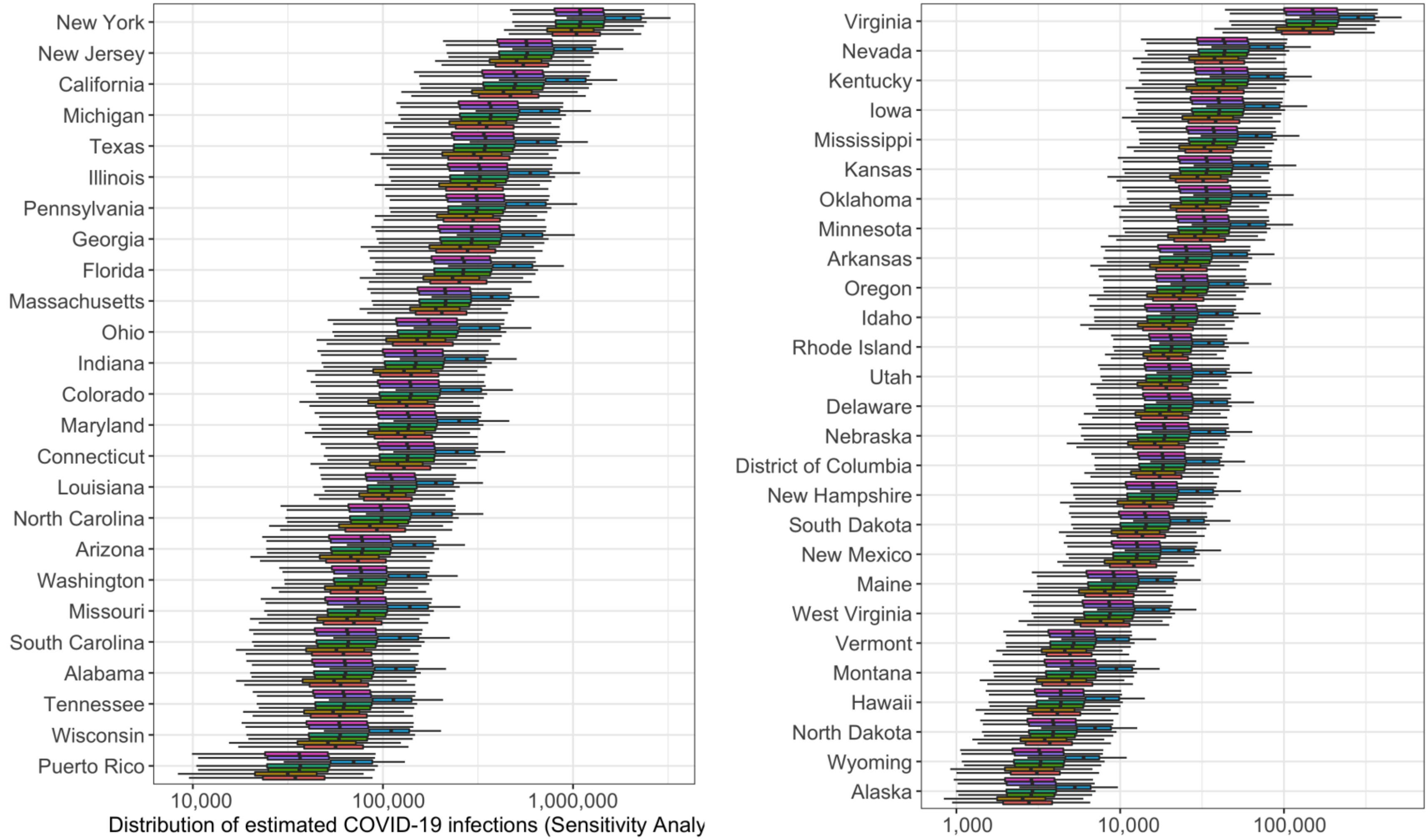

For each state we drew 10^4 Monte Carlo samples from the distribution of estimated SARS-CoV-2 infections, under each of the seven scenarios considered. For each state, the scenarios summarized as boxplots are presented from bottom to top: salmon (bottom) 1, mustard 2, green 3, teal 4, blue 5, purple 6, pink 7. The lower and upper whiskers of each boxplot corresponds to the 0.025 and 0.975 quantiles, respectively, and the lower and upper hinge to the 0.25 and 0.75 quantiles. The heavy vertical line (centre) is the median of sampled values. Descriptions of each scenario are given in Supplementary Table 2.

Supplementary Figure 3. Static prior distributions for probabilistic bias analysis

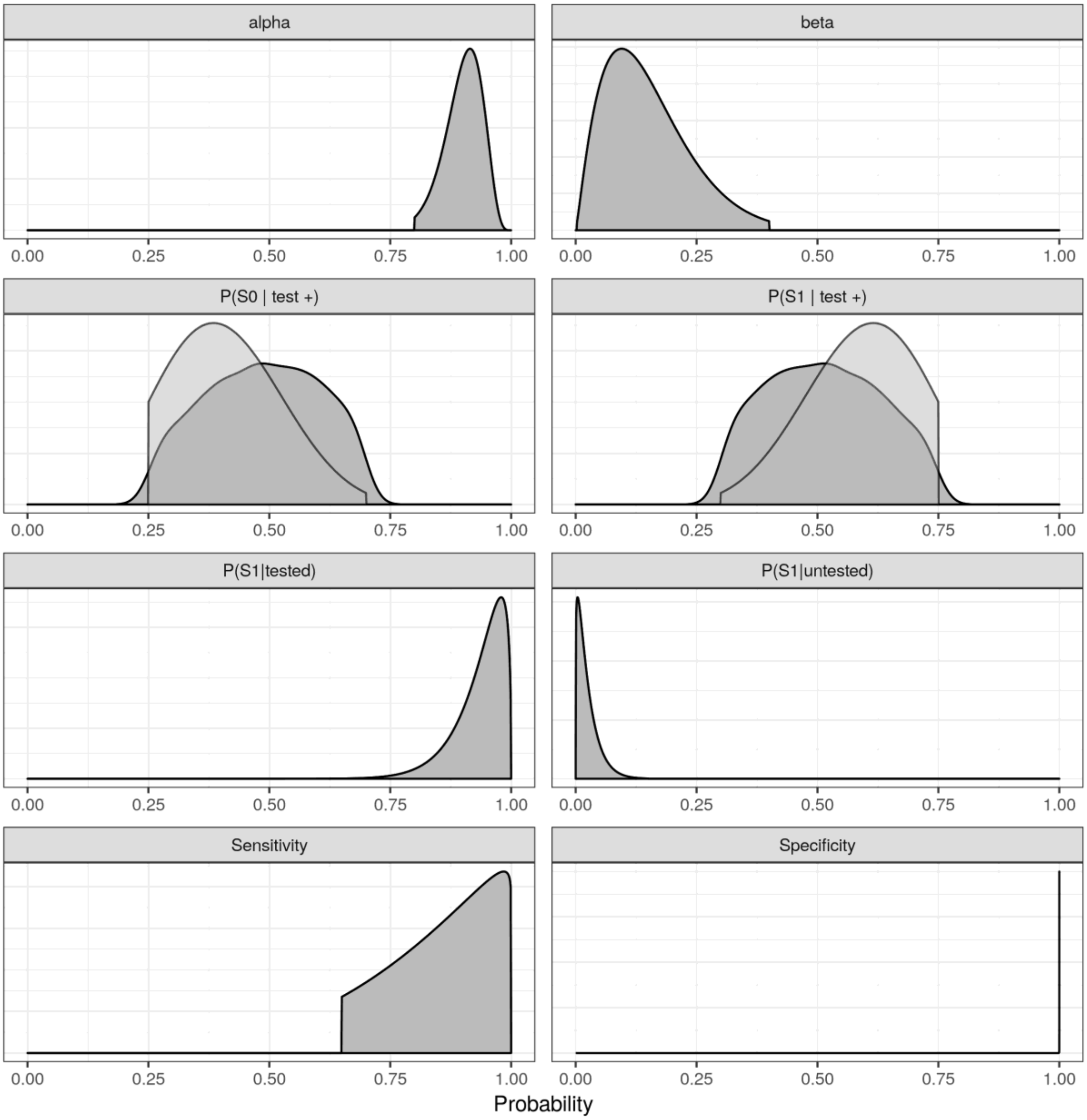

We assumed specificity ranged from 0.9998 to 1.000. Plots for P($\underline{S_0}$|test +) and P($\underline{S_1}$|test +) show the prior distribution before Bayesian melding (lighter grey foreground) and after Bayesian melding (dark grey background). The post-Bayesian melding densities are kernel density estimates from 10^5 sampled values, because the post-Bayesian melding distributions do not have an analytic form.

Supplementary Figure 4. Probability of testing positive among those tested in each state by date

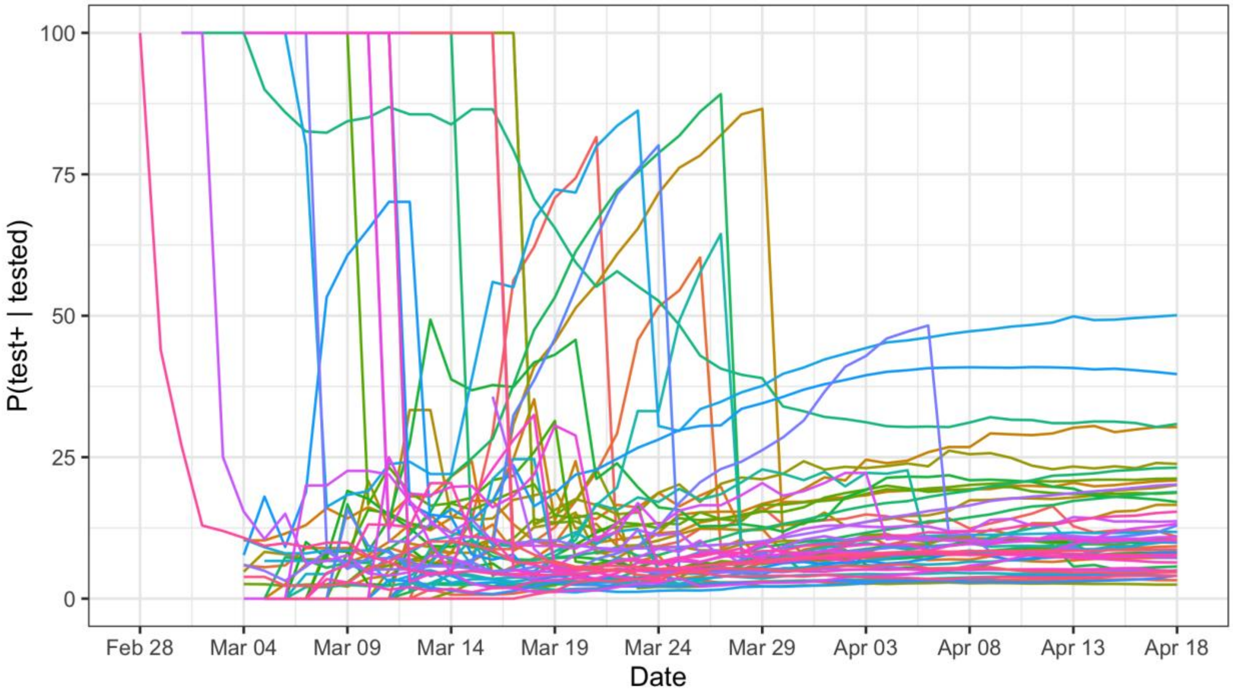

Each line is P(test+ |tested) in a state by date. Prior to late March, 2020, the percentage of the population tested was less than 0.6%, and P(test+ |tested) was highly variable over time.

Supplementary Figure 5. Probability of testing positive among those tested in each state by date restricting to dates when the percentage of the population tested was at least 0.6%

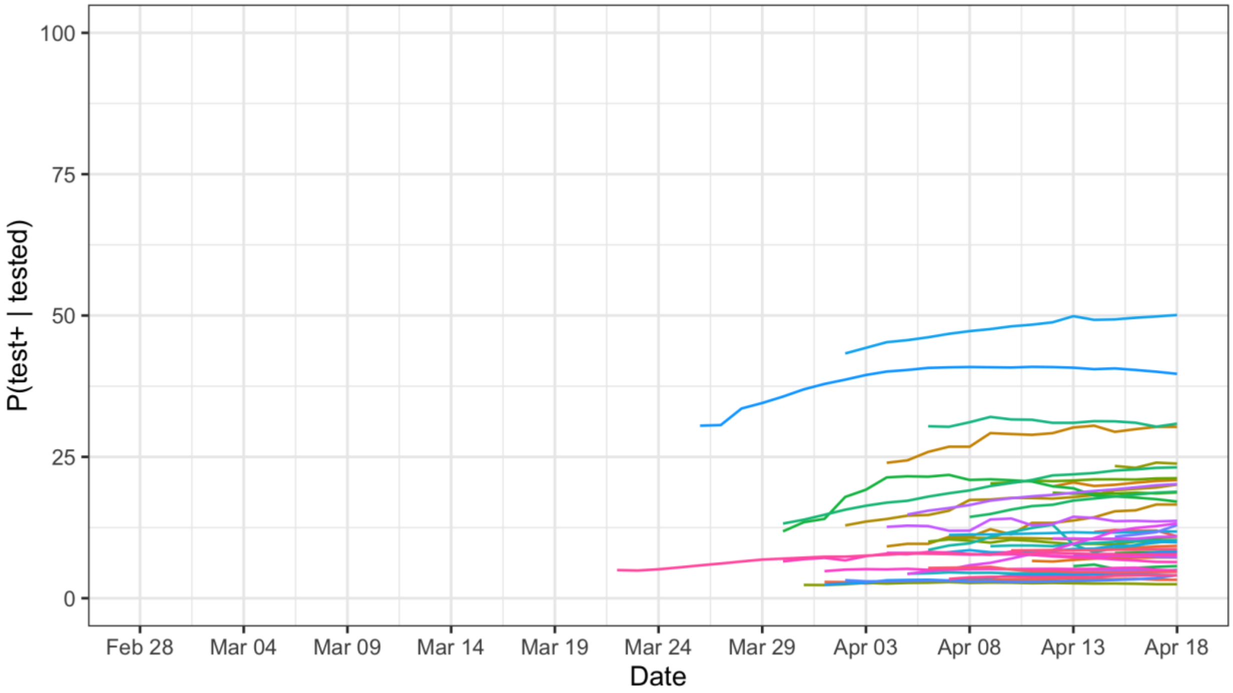

Each line is P(test+ |tested) in a state by date. Starting in late March, 2020, the percentage of the population tested was at least 0.6% in each state, and P(test+ |tested) was substantially less variable over time.

Supplementary Figure 6. Visual description of probabilistic bias analysis sampling algorithm

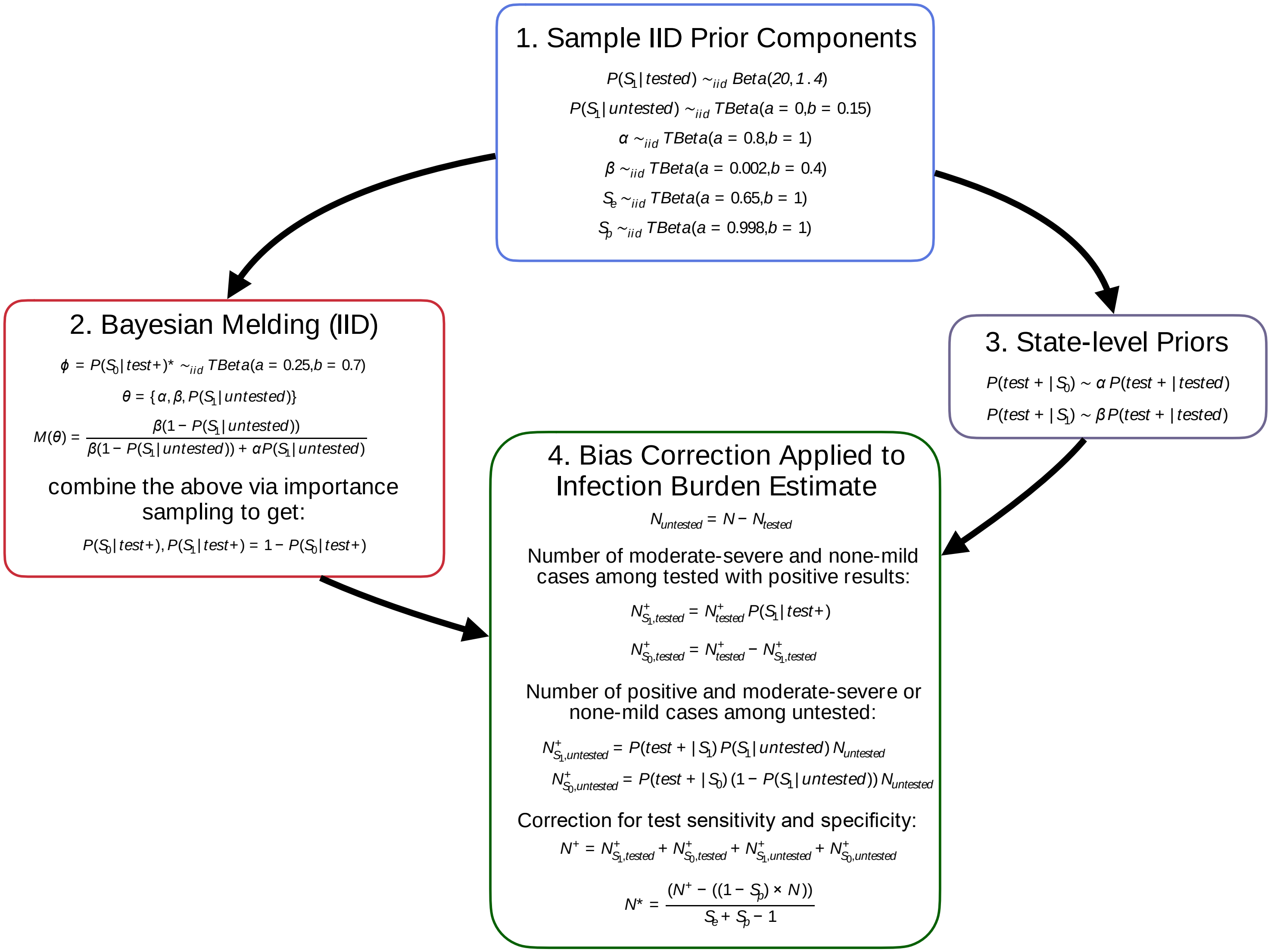

TBeta(a,b) refers to a truncated Beta distribution with lower and upper bounds of truncation as *a*,*b* (see Supplementary Table 2 for the full parameterization).

Supplementary Table 1. State-level recommendations for SARS-CoV-2 testing in the general population by symptom status

State	Individuals with moderate and/or severe symptoms	Individuals with mild symptoms and/or asymptomatic	Reference number
Alaska	Yes	Yes*	1
Alabama	Yes	No	2
Arkansas	Yes	Yes**	3
Arizona	Yes	No	4
California	Yes	Yes**	5
Colorado	Yes	No	6
Connecticut	Yes	No	7
District of Columbia	Yes	No	8
Delaware	Yes	Yes*	9
Florida	Yes	Yes*	10
Georgia	Yes	Yes*	11
Hawaii	Yes	No	12
Iowa	Yes	No	13
Idaho	Yes	No	14
Illinois	Yes	No	15
Indiana	Yes	No	16
Kansas	Yes	No	17
Kentucky	Yes	Yes**	18
Louisiana	Yes	Yes*	19
Massachusetts	Yes	No	20
Maryland	Yes	No	21
Maine	Yes	No	22
Michigan	Yes	Yes*	23
Minnesota	Yes	Yes**	24
Missouri	Yes	No	25
Mississippi	Yes	No	26
Montana	Yes	No	27
North Carolina	Yes	No	28
North Dakota	Yes	Yes*	29
Nebraska	Yes	No	30
New Hampshire	Yes	No	31
New Jersey	Yes	No	32

New Mexico	Yes	Yes*	33
Nevada	Yes	No	34
New York	Yes	No	35,36
Ohio	Yes	Yes**	37
Oklahoma	Yes	Yes*	38
Oregon	Yes	No	39
Pennsylvania	Yes	No	40
Rhode Island	Yes	Yes*	41
South Carolina	Yes	Yes**	42
South Dakota	Yes	No	43
Tennessee	Yes	Yes*	44
Texas	Yes	No	45
Utah	Yes	Yes*	46
Virginia	Yes	No	47
Vermont	Yes	Yes*	48
Washington	Yes	No	49
Wisconsin	Yes	Yes*	50
West Virginia	Yes	No	51
Wyoming	Yes	Yes**	52

Each source was originally accessed on April 26-27, 2020. PDFs of each site from these dates are available here: https://tinyurl.com/ya62kr2q

Recommendations vary for high risk (e.g., 65 and older) and priority groups (e.g., contacts of confirmed COVID-19 cases, health providers and first responders). These categories reflect state-level testing recommendations as of 4/26 for the general population. Some states recommend testing for those with any symptoms, but only if supplies are available after testing high risk groups. Therefore, the categories included here may overestimate who is tested when testing capacity is limited or underestimate who is tested if health providers refer patients who do not meet recommended criteria to commercial or private labs. In general, moderate and/or severe category includes hospitalized patients or symptoms requiring medical attention, as noted in testing recommendations. In general, mild symptoms include any symptoms or mild symptoms, generally requiring no medical attention, as noted in testing recommendations. Very few state testing recommendations include asymptomatic individuals. CDC recommendations include any symptomatic individuals as a low testing priority but also note that individuals with mild symptoms may not need testing; this is categorized as "No" recommendation for testing among all mildly symptomatic/asymptomatic individuals. These categories and dates are based on the best available information from state coronavirus websites, state press releases or related news coverage, health alert networks (HAN), or state government social media accounts.

*Testing was expanded to include this population during the study period. Approximate dates when states expanded testing recommendations are as follows: MI (4/14); ND (3/24); NM (4/1); RI (4/1); TN (4/18 for biggest expansion to anyone who wants testing); UT (4/10); VT (3/27); DE (3/31); GA (4/15); AK (4/8); FL (early April); LA (3/18); OK (4/1); WI (4/16)

** Testing was expanded to include this population after the study period. Approximate dates when states expanded testing recommendations are as follows: CA (4/19); AR (4/24); KY (4/27); MN (4/23); OH (4/22); SC (4/22); WY (4/22)

Supplementary Table 2. Distributions under Scenarios Considered for Sensitivity Analyses

Scenario	Distribution(s) Affected	Minimum (lower bound)	Mean	Maximum (upper bound)	Shape 1	Shape 2
1. Alpha shift down 1	α	0.500	0.850	1.000	66.884	11.803
2. Alpha shift down 2	α	0.250	0.750	1.000	87.141	29.040
3. P(S_1|tested) shift down	P(S_1|tested)	0.000	0.800	1.000	46.095	11.524
4. P(S_1|untested) shift upper bound to 25%	P(S_1|untested)	0.000	0.025	0.250	1.178	45.969
5. Upward shift β	β	0.250	0.250	0.600	5.537	16.611
6. Mild Correlation (0.2)	α, β	Unchanged	Unchanged	Unchanged	Unchanged	Unchanged
7. High Correlation (0.8)	α, β	Unchanged	Unchanged	Unchanged	Unchanged	Unchanged

Scenarios 6 and 7 (mild and high correlation between α, β) affect the joint distribution of α, β by introducing correlation (non-independence) but do not change the marginal distributions. Correlation was introduced at the specified level by simulating from a Gaussian copula using correlation of 0.2 and 0.8 for each scenario, respectively.

Supplementary Table 3. Quality of state-level test reports according to COVID Tracking Project

Grade	States
A	Alaska, Arkansas, Colorado, Connecticut, District of Columbia, Delaware, Florida, Georgia, Hawaii, Iowa, Idaho, Illinois, Kentucky, Louisiana, Maryland, Maine, Minnesota, Missouri, Mississippi, Montana, North Carolina, Nebraska, New Jersey, New Mexico, Nevada, New York, Oregon, Pennsylvania, South Dakota, Tennessee, Texas, Utah, Virginia, Vermont, Wisconsin, Wyoming
B	Alabama, Arizona, California, Indiana, Kansas, Massachusetts, Michigan, North Dakota, New Hampshire, Ohio, Oklahoma, Rhode Island, South Carolina, West Virginia
C	Washington, Puerto Rico

The COVID Tracking Project assigned grades to each state based on four criteria: 1) reporting positives reliably, 2) Reporting negatives sometimes, 3) reporting negatives reliably, and 4) reporting all commercial tests. States meeting all four criteria received an “A” grade, those meeting three criteria received a “B” grade, and those meeting two criteria received a “C” grade.

Supplementary Table 4. State-specific prior distributions for probabilistic bias analysis

State	P(test+|S_1, untested)			P(test+|S_0, untested)		
	Minimum	Median	Maximum	Minimum	Median	Maximum
Alabama	0.088	0.099	0.108	0.000	0.005	0.035
Alaska	0.026	0.03	0.032	0.000	0.001	0.01
Arizona	0.074	0.084	0.091	0.000	0.004	0.029
Arkansas	0.058	0.065	0.071	0.000	0.003	0.023
California	0.087	0.099	0.107	0.000	0.005	0.034
Colorado	0.167	0.19	0.206	0.001	0.01	0.066
Connecticut	0.242	0.275	0.299	0.001	0.014	0.096
Delaware	0.133	0.151	0.164	0.000	0.008	0.053
District of Columbia	0.161	0.183	0.199	0.001	0.009	0.064
Florida	0.082	0.093	0.101	0.000	0.005	0.033
Georgia	0.190	0.216	0.235	0.001	0.011	0.076
Hawaii	0.020	0.022	0.024	0.000	0.001	0.008
Idaho	0.080	0.091	0.098	0.000	0.005	0.032
Illinois	0.170	0.193	0.210	0.001	0.010	0.067
Indiana	0.150	0.17	0.185	0.000	0.009	0.059
Iowa	0.088	0.099	0.108	0.000	0.005	0.035
Kansas	0.081	0.092	0.100	0.000	0.005	0.032
Kentucky	0.066	0.075	0.081	0.000	0.004	0.026
Louisiana	0.137	0.155	0.169	0.000	0.008	0.054
Maine	0.045	0.052	0.056	0.000	0.003	0.018
Maryland	0.151	0.171	0.186	0.000	0.009	0.060
Massachusetts	0.186	0.211	0.229	0.001	0.011	0.074
Michigan	0.247	0.281	0.305	0.001	0.014	0.098
Minnesota	0.040	0.045	0.049	0.000	0.002	0.016
Mississippi	0.082	0.093	0.101	0.000	0.005	0.033
Missouri	0.082	0.093	0.101	0.000	0.005	0.033
Montana	0.032	0.037	0.04	0.000	0.002	0.013
Nebraska	0.067	0.076	0.082	0.000	0.004	0.026
Nevada	0.094	0.107	0.117	0.000	0.005	0.037
New Hampshire	0.079	0.090	0.098	0.000	0.005	0.031
New Jersey	0.401	0.455	0.495	0.001	0.023	0.159
New Mexico	0.038	0.044	0.047	0.000	0.002	0.015
New York	0.317	0.361	0.392	0.001	0.018	0.126
North Carolina	0.064	0.073	0.080	0.000	0.004	0.026
North Dakota	0.033	0.037	0.040	0.000	0.002	0.013
Ohio	0.101	0.115	0.125	0.000	0.006	0.040
Oklahoma	0.058	0.066	0.071	0.000	0.003	0.023
Oregon	0.039	0.045	0.048	0.000	0.002	0.016
Pennsylvania	0.161	0.183	0.199	0.001	0.009	0.064
Puerto Rico	0.082	0.093	0.101	0.000	0.005	0.032
Rhode Island	0.109	0.124	0.135	0.000	0.006	0.043
South Carolina	0.087	0.099	0.108	0.000	0.005	0.035
South Dakota	0.106	0.120	0.131	0.000	0.006	0.042
Tennessee	0.060	0.068	0.074	0.000	0.003	0.024
Texas	0.083	0.094	0.102	0.000	0.005	0.033

Utah	0.039	0.044	0.048	0.000	0.002	0.016
Vermont	0.051	0.058	0.063	0.000	0.003	0.020
Virginia	0.123	0.140	0.152	0.000	0.007	0.049
Washington	0.072	0.081	0.089	0.000	0.004	0.028
West Virginia	0.032	0.037	0.040	0.000	0.002	0.013
Wisconsin	0.069	0.079	0.086	0.000	0.004	0.028
Wyoming	0.037	0.042	0.046	0.000	0.002	0.015

Supplementary Information References

1. Alaska Department of Health and Social Services. Public Health Alert Network (PHAN). http://dhss.alaska.gov/dph/Epi/Pages/phan/default.aspx.
2. Alabama Department of Public Health. Healthcare Providers | Alabama Department of Public Health (ADPH). https://www.alabamapublichealth.gov/covid19/healthcare.html.
3. Carroll, S. Arkansas plans surge in COVID-19 testing; deaths in state up to 45. *KATV* https://katv.com/news/coronavirus/arkansas-plans-surge-in-covid-19-testing-deaths-in-state-up-to-45 (2020).
4. Arizona Department of Health Services. *Arizona State Public Health Laboratory Testing Matrix*. https://www.azdhs.gov/documents/preparedness/epidemiology-disease-control/infectious-diseases-services/coronavirus/arizona-state-public-health-laboratory-testing-matrix.pdf (2020).
5. California Department of Public Health. Expanding Access to Testing: Updated Guidance on Prioritization for COVID-19 Testing. https://www.cdph.ca.gov/Programs/CID/DCDC/Pages/COVID-19/Expanding-Access-to-Testing-Updated-Guidance-on-Prioritization-for-COVID-19-Testing.aspx (2020).
6. Colorado Department of Public Health and Environment. Testing for COVID-19 | Colorado COVID-19 Updates. https://covid19.colorado.gov/are-you-sick/testing-for-covid-19.
7. State of Connecticut Department of Public Health. *Updated Guidance for COVID-19: PPE Conservation and Testing Strategies*. https://portal.ct.gov/-/media/Coronavirus/20200324-Update_PPE_Testing_Info.pdf?la=en (2020).

8. Government of the District of Columbia. District of Columbia COVID-19 Testing Sites | coronavirus. https://coronavirus.dc.gov/testing.
9. Delaware Health and Social Services. *Coronavirus Disease 2019 (COVID-19) FAQs for Testing*. https://coronavirus.delaware.gov/wp-content/uploads/sites/177/2020/04/Coronavirus-Testing-FAQ_4.9.20.pdf (2020).
10. Florida Health. Health Care Providers. *Florida Department of Health COVID-19 Outbreak* https://floridahealthcovid19.gov/health-care-providers/.
11. Georgia Department of Public Health. COVID-19: Guidance for Healthcare Professionals. *Georgia Department of Public Health* https://dph.georgia.gov/covid-19-guidance-healthcare-professionals.
12. State of Hawai'i Department of Health. Criteria for Evaluating and Testing Persons for COVID-19. https://health.hawaii.gov/coronavirusdisease2019/for-clinicians/evaluating-puis/.
13. Iowa Department of Public Health. Novel Coronavirus - Healthcare. https://idph.iowa.gov/Emerging-Health-Issues/Novel-Coronavirus/Healthcare.
14. Government of Idaho, A. Frequently Asked Questions. *Novel Coronavirus (COVID-19)* https://coronavirus.idaho.gov/frequently-asked-questions/ (2020).
15. Illinois Department of Public Health. *COVID-19 Testing Guidance*. https://www.dph.illinois.gov/sites/default/files/COVID19/COVID-19_TestingGuidance.pdf.
16. Indiana State Department of Health. *COVID-19 Testing Information*. https://www.coronavirus.in.gov/files/IN_COVID-19_testing_4.23.2020.pdf (2020).

17. Kansas Department of Health and Environment. KDHE COVID-19. https://ks-kdhecovid19.civicplus.com/faq.aspx?TID=17.

18. Key, J. & Mazade, L. Kentucky Gov. Beshear announces drive-thru testing sites open to public; 185 total deaths in state. *The Enquirer* https://www.cincinnati.com/story/news/2020/04/22/coronavirus-kentucky-governor-andy-beshear-press-conference-covid-19/3004434001/ (2020).

19. Louisiana Department of Environmental Quality. COVID-19 FAQs - 3.18.2020. https://www.deq.louisiana.gov/page/covid19-faqs-3182020 (2020).

20. Brown, C. M., Madoff, L. & Smole, S. *Testing of Persons with Suspect COVID-19*. https://www.mass.gov/doc/covid-19-pui-criteria/download (2020).

21. Maryland Department of Health. Resources for Health Care Professionals. https://coronavirus.maryland.gov/pages/provider-resources.

22. Bennett, S. *Maine Health Alert Network (HAN) System Public Health Advisory*. https://www.maine.gov/dhhs/mecdc/infectious-disease/epi/airborne/documents/COVID-19-Prioritization-Of-Testing-And-Discontinuation-Of-Isolation.pdf (2020).

23. State of Michigan. *Michigan Interim COVID-19 Person Under Investigation (PUI) Case Report Form*. https://www.michigan.gov/documents/coronavirus/MDHHS_PUI_Form_Fillable_v04.09.2020_686599_7.pdf (2020).

24. Minnesota Department of Health. *Health Advisory: Expanded SARS-CoV-2 Testing*. https://www.health.state.mn.us/communities/ep/han/2020/apr23testing.pdf (2020).

25. Williams, R. W. *Update: New Criteria to Guide Evaluation and Laboratory Testing for COVID-19 at the Missouri State Public Health Laboratory*. https://health.mo.gov/emergencies/ert/alertsadvisories/pdf/update42220.pdf (2020).
26. Mississippi State Department of Health. *Priority COVID-19 Testing at the Mississippi Public Health Laboratory*. https://msdh.ms.gov/msdhsite/_static/resources/8538.pdf (2020).
27. State of Montana. Coronavirus. *Coronavirus Disease 2019 (COVID-19)* https://dphhs.mt.gov/publichealth/cdepi/diseases/coronavirusmt.
28. North Carolina Department of Health and Human Services. NC DHHS COVID-19: Testing. https://covid19.ncdhhs.gov/about-covid-19/testing.
29. North Dakota Health. *Updated COVID-19 Testing Guidance*. https://www.health.nd.gov/sites/www/files/documents/Files/MSS/HAN/Updated_COVID-19_Testing_Guidance.pdf (2020).
30. Nebraska Department of Health and Human Services. *Coronavirus Disease 2019 (COVID-19): General Guidance and Testing Information for Nebraskans*. http://dhhs.ne.gov/Documents/COVID-19%20Guidance%20to%20Public%20and%20Testing.pdf (2020).
31. New Hampshire Division of Public Health Services. *Coronavirus Disease 2019 (COVID-19) Outbreak, Update # 12*. https://www.dhhs.nh.gov/dphs/cdcs/alerts/documents/covid-19-update12.pdf (2020).
32. New Jersey Department of Health. *COVID-19 Frequently Asked Questions*. https://www.nj.gov/health/cd/documents/topics/NCOV/COVID19_public_faqs.pdf (2020).

33. New Mexico Department of Health. What are the symptoms of COVID-19 and should I be tested? https://cv.nmhealth.org/public-health-screening-and-testing/location-details/.

34. Nevada Health Response. *COVID-19 Testing Information for Health Care Providers*. https://nvhealthresponse.nv.gov/wp-content/uploads/2020/03/03.24-COVID-19-Testing-Information-for-Health-Care-Providers.pdf.

35. New York State. COVID-19 Testing. *Department of Health* https://coronavirus.health.ny.gov/covid-19-testing.

36. New York City Health. COVID-19: Data Summary - NYC Health. https://www1.nyc.gov/site/doh/covid/covid-19-data.page.

37. Ohio Government. *Updated COVID-19 Testing Guidance April 22, 2020*. https://coronavirus.ohio.gov/wps/portal/gov/covid-19/healthcare-providers-and-local-health-districts/for-healthcare-providers/updated-covid-19-testing-guidance-april-22-2020 (2020).

38. @HealthyOklahoma (Oklahoma Department of Health). 1/2 #OSDH Commissioner of Health Gary Cox issued a letter notifying county health departments today that all #COVID19 testing centers and providers are to loosen requirements for Oklahomans to be tested. *Twitter* https://twitter.com/HealthyOklahoma/status/1245494904823873538 (2020).

39. Oregon Health Authority. *COVID-19 testing guidance for health care providers*. https://sharedsystems.dhsoha.state.or.us/DHSForms/Served/le2267.pdf (2020).

40. Pennsylvania Department of Health. COVID-19 Symptoms and Testing. *Department of Health* https://www.health.pa.gov/topics/disease/coronavirus/Pages/Symptoms-Testing.aspx.

41. State of Rhode Island Department of Health. COVID-19 Testing for the General Public: Department of Health. https://health.ri.gov/covid/testing/.
42. South Carolina Department of Health and Environmental Control. *Updated reporting criteria for 2019 novel coronavirus disease (COVID-19)*. https://www.scdhec.gov/sites/default/files/media/document/10458-DHU-04-22-2020-COVID-19_0.pdf (2020).
43. South Dakota Department of Health. *COVID-19 Frequently Asked Questions*. https://doh.sd.gov/documents/COVID19/covid_faqs.pdf.
44. Tennessee Office of the Governor. Get Tested. https://www.tn.gov/governor/covid-19/get-tested.html.
45. Texas Health and Human Services. Information for Hospitals & Healthcare Professionals. https://www.dshs.state.tx.us/coronavirus/healthprof.aspx#eval.
46. Utah Government. Testing Locations | coronavirus. https://coronavirus.utah.gov/testing-locations/.
47. Virginia Department of Health. COVID-19 Testing – Coronavirus. https://www.vdh.virginia.gov/coronavirus/covid-19-testing/, https://www.vdh.virginia.gov/coronavirus/covid-19-testing/.
48. Vermont Department of Health. Health Care Professionals. *Vermont Department of Health* https://www.healthvermont.gov/response/infectious-disease/novel-coronavirus-covid-19-health-care-professionals (2020).

49. Washington State Department of Health. Testing for COVID-19. https://www.doh.wa.gov/Emergencies/NovelCoronavirusOutbreak2020COVID19/TestingforCOVID19.

50. Wisconsin Department of Health Services. COVID-19: Health Alert #5: DHS Now Recommends Health Care Providers Test All Patients with Acute Respiratory Illness for COVID-19. *Wisconsin Department of Health Services* https://content.govdelivery.com/accounts/WIDHS/bulletins/286efb3 (2020).

51. West Virginia Department of Health and Human Resources. *Coronavirus disease 2019 (COVID-19) testing information for healthcare providers*. https://dhhr.wv.gov/COVID-19/Documents/hcp/COVID19-Testing-Information-for-Healthcare-Providers.pdf.

52. Wyoming Department of Health. *State Health Advisory Supplement Updated Guidance for Coronavirus Disease 2019 (COVID-19) Testing*. https://health.wyo.gov/wp-content/uploads/2020/04/Coronavirus_Disease_2019_HAN_10.1_4.22.20.pdf (2020).
