# Supplementary material for: Substantial underestimation of SARS-CoV-2 infection in the United States
Source: Nat Commun. 2020 Sep 9;11:4507. doi: 10.1038/s41467-020-18272-4 (PMC7481226; doi:10.1038/s41467-020-18272-4)
Supplement: Supplementary file 3 — Reporting Summary [file 41467_2020_18272_MOESM3_ESM.pdf]

nature research

Corresponding author(s): Jade Benjamin-Chung

Last updated by author(s): Aug 11, 2020

Reporting Summary

Nature Research wishes to improve the reproducibility of the work that we publish. This form provides structure for consistency and transparency in reporting. For further information on Nature Research policies, see our Editorial Policies and the Editorial Policy Checklist.

Statistics

For all statistical analyses, confirm that the following items are present in the figure legend, table legend, main text, or Methods section.

n/a	Confirmed	
x		The exact sample size (*n*) for each experimental group/condition, given as a discrete number and unit of measurement
x		A statement on whether measurements were taken from distinct samples or whether the same sample was measured repeatedly
x		The statistical test(s) used AND whether they are one- or two-sided<br>*Only common tests should be described solely by name; describe more complex techniques in the Methods section.*
	x	A description of all covariates tested
	x	A description of any assumptions or corrections, such as tests of normality and adjustment for multiple comparisons
	x	A full description of the statistical parameters including central tendency (e.g. means) or other basic estimates (e.g. regression coefficient) AND variation (e.g. standard deviation) or associated estimates of uncertainty (e.g. confidence intervals)
x		For null hypothesis testing, the test statistic (e.g. *F*, *t*, *r*) with confidence intervals, effect sizes, degrees of freedom and *P* value noted<br>*Give P values as exact values whenever suitable.*
	x	For Bayesian analysis, information on the choice of priors and Markov chain Monte Carlo settings
x		For hierarchical and complex designs, identification of the appropriate level for tests and full reporting of outcomes
x		Estimates of effect sizes (e.g. Cohen's *d*, Pearson's *r*), indicating how they were calculated

Our web collection on statistics for biologists contains articles on many of the points above.

Software and code

Policy information about availability of computer code

Data collection	The data that support the findings of this study are available from the COVID Tracking Project (https://covidtracking.com/).
Data analysis	All data curation/collection, analyses, and figures were produced with the R programming language version 3.6.3 (Holding the Windsock); all code is available at the GitHub repository https://github.com/jadebc/covid19-infections/releases/tag/NatureComms, and a permanent archive is available at https://doi.org/10.5281/zenodo.3976252

For manuscripts utilizing custom algorithms or software that are central to the research but not yet described in published literature, software must be made available to editors and reviewers. We strongly encourage code deposition in a community repository (e.g. GitHub). See the Nature Research guidelines for submitting code & software for further information.

Data

Policy information about availability of data

All manuscripts must include a data availability statement. This statement should provide the following information, where applicable:

- Accession codes, unique identifiers, or web links for publicly available datasets
- A list of figures that have associated raw data
- A description of any restrictions on data availability

The data that support the findings of this study are available from the COVID Tracking Project (https://covidtracking.com/).

April 2020

Field-specific reporting

Please select the one below that is the best fit for your research. If you are not sure, read the appropriate sections before making your selection.

☐ Life sciences ☒ Behavioural & social sciences ☐ Ecological, evolutionary & environmental sciences

For a reference copy of the document with all sections, see nature.com/documents/nr-reporting-summary-flat.pdf

Behavioural & social sciences study design

All studies must disclose on these points even when the disclosure is negative.

Study description	The objective of this study is to estimate the total number of SARS-CoV-2 infections in the U.S. from February 28 to April 18, 2020 correcting for incomplete testing and imperfect test accuracy. This study used a semi-Bayesian quantitative bias analysis and publicly available data sets.
Research sample	Data used on COVID-19 cases is provided by the COVID Tracking Project available at https://covidtracking.com/. Data on state populations is provided by the United States Census available at https://www.census.gov/data/tables/time-series/demo/popest/2010s-state-total.html#par_textimage.
Sampling strategy	No sample size calculation was performed because the data sources used represents the best possible sources of publicly available data to our knowledge.
Data collection	This study used publicly available from the COVID Tracking Project (https://covidtracking.com/).
Timing	COVID-19 data was collected from the COVID Tracking Project from February 28 to April 18, 2020, but can easily be updated with provided code.
Data exclusions	No data were excluded from analysis.
Non-participation	This is not applicable because this study used publicly available from the COVID Tracking Project (https://covidtracking.com/). This source compiles data from local public health authority websites.
Randomization	This is not applicable because this study used publicly available from the COVID Tracking Project (https://covidtracking.com/). This source compiles data from local public health authority websites.

Reporting for specific materials, systems and methods

We require information from authors about some types of materials, experimental systems and methods used in many studies. Here, indicate whether each material, system or method listed is relevant to your study. If you are not sure if a list item applies to your research, read the appropriate section before selecting a response.

Materials & experimental systems

n/a	Involved in the study	
☒	☐	Antibodies
☒	☐	Eukaryotic cell lines
☒	☐	Palaeontology and archaeology
☒	☐	Animals and other organisms
☒	☐	Human research participants
☒	☐	Clinical data
☒	☐	Dual use research of concern

Methods

n/a	Involved in the study	
☒	☐	ChIP-seq
☒	☐	Flow cytometry
☒	☐	MRI-based neuroimaging
